# Abundance, species composition of microzooplankton from the coastal waters of Port Blair, South Andaman Island

**DOI:** 10.1186/2046-9063-8-20

**Published:** 2012-08-31

**Authors:** S Sai Elangovan, M Arun Kumar, R Karthik, R Siva Sankar, R Jayabarathi, G Padmavati

**Affiliations:** 1 Department of Ocean Studies and Marine Biology, Pondicherry University, Port Blair, Andaman, 744 103, India; 2 Department of Ecology and Environmental Sciences, Pondicherry University, Puducherry, 605 014, India

**Keywords:** Microzooplankton, Tintinnids, Plankton distribution, South Andaman Sea

## Abstract

**Background:**

Microzooplankton consisting of protists and metazoa <200 μm. It displays unique feeding mechanisms and behaviours that allow them to graze cells up to five times their own volume. They can grow at rates which equal or exceed prey growth and can serve as a viable food source for metazoans. Moreover, they are individually inconspicuous, their recognition as significant consumers of oceanic primary production. The microzooplankton can be the dominant consumers of phytoplankton production in both oligo- and eutrophic regions of the ocean and are capable of consuming >100% of primary production.

**Results:**

The microzooplankton of the South Andaman Sea were investigated during September 2011 to January 2012. A total of 44 species belong to 19 genera were recorded in this study. Tintinnids made larger contribution to the total abundance (34%) followed in order by dinoflagellates (24%), ciliates (20%) and copepod nauplii (18%). Foraminifera were numerically less (4%). Tintinnids were represented by 20 species belong to 13 genera, Heterotrophic dinoflagellates were represented by 17 species belong to 3 genera and Ciliates comprised 5 species belong to 3 genera. *Eutintinus tineus, Tintinnopsis cylindrical, T. incertum, Protoperidinium divergens, Lomaniella oviformes, Strombidium minimum* were the most prevalent microzooplankton. Standing stock of tintinnids ranged from 30–80 cells.L^-1^ and showed a reverse distribution with the distribution of chlorophyll *a* relatively higher species diversity and equitability was found in polluted harbour areas.

**Conclusions:**

The change of environmental variability affects the species composition and abundance of microzooplankton varied spatially and temporarily. The observations clearly demonstrated that the harbor area differed considerably from other area in terms of species present and phytoplankton biomass. Further, the phytoplankton abundance is showed to be strongly influenced by tintinnid with respect to the relationship of prey–predator. Consequently, further investigation on microzooplankton grazing would shed light on food web dynamics.

## Background

Microzooplankton or microplankton (20–200 μm) are heterotrophic [1]. They play a significant role in energy transfer through marine pelagic food web and hence their ecology and dynamics received considerable attention in recent times [2]. Microzooplankton are significant grazers of Phytoplankton compared to Mesozooplankton [3,4]. They are comprised of tintinnids, dinoflagellates, ciliates and crustacean nauplii and are capable of exploiting pico and nanoplankton (2–20 μm) and in turn are underutilized by other large zooplankton [5,6].

Despite several studies on these organisms from other areas, our knowledge on their ecobiology from the Indian Ocean is limited [7-10]. There is apparently no study from coastal waters of south Andaman, in order to understand how the species in these vicinages interact and how far their distributions overlap. Hence, to fill these lacunae, it is considered necessary to undertake an in-depth study of microzooplankton ecology from the coastal waters of South Andaman.

## Results

**Figure 1:**
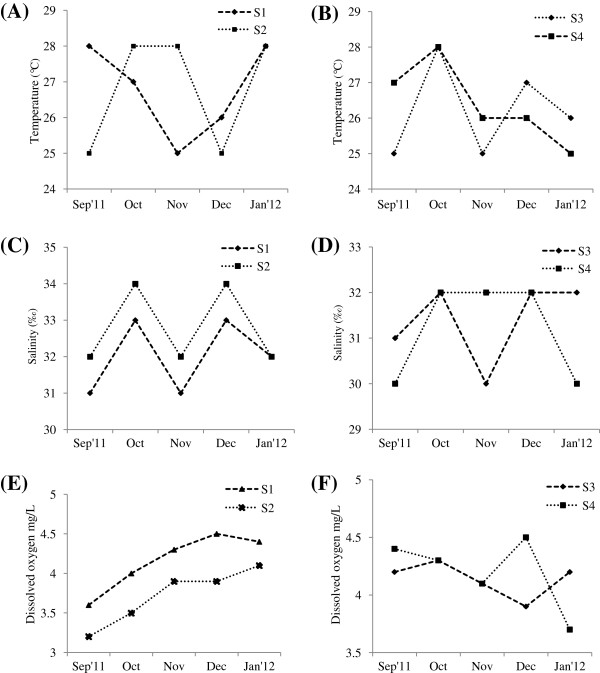
Variations in physico-chemical parameters during September’11-January’12.

**Figure 2:**
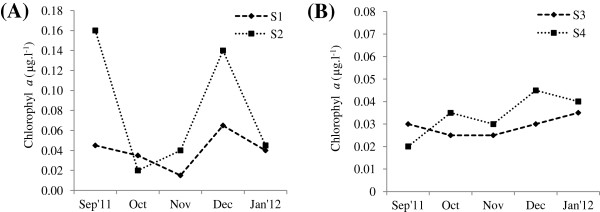
**Variations in Chlorophyll *a *concentration during September’11- January’12.**

During the study period, water temperature ranged from 25- 28°C at all stations. Salinity ranged from 30 to 34 ppt and it was recorded high during October and December at all stations. The dissolved oxygen varied from 3.2 mg/l- 4.5 mg/l. High values of Dissolved oxygen value was recorded during December at St.1and St.4. Environmental parameters such as surface water temperature and salinity were recorded low during monsoon month (September) (Figure 1). Chlorophyl *a* concentration varied from 0.02 - 0.16 μg l^-1^. Higher values of Chl *a* (0.16 μg l^-1^) was recorded during September’11 at St.2 was due to the diatoms bloom *Coscinodiscus centralis* followed by 0.14 μg l^-1^ during December’11 was due to the bloom of *Rhizosolenia alata* at St.2 (Figure 2).

### Microzooplankton density and composition

**Figure 3:**
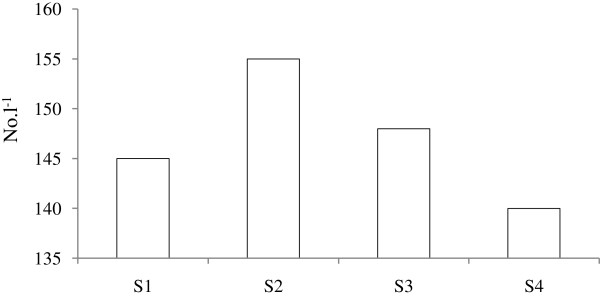
Abundance of microzooplankton during September’11-January ’12.

**Figure 4:**
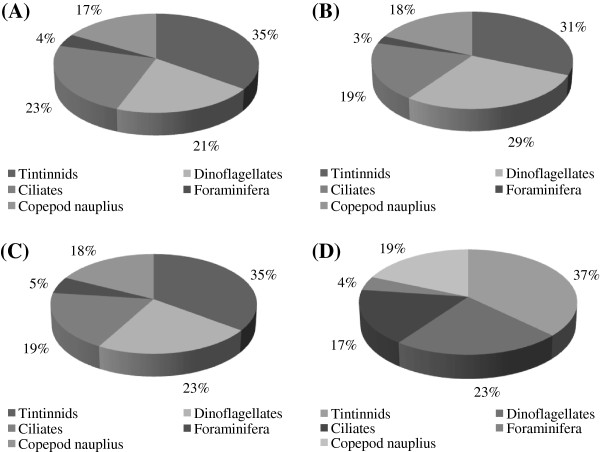
Percentage composition of Microzooplankton at S1, S2, S3 and S4.

Microzooplankton density was higher (*t*-test; *p* <0.05) in St. 2 compared to other study area. The overall mean abundance was higher in October’11 when high salinity and temperature were recorded. The copepod nauplius only belongs to the category of multicellular organisms and others are known to be as unicelluar organisms. The unicellular organism of microzooplankton was recorded maximum density (126 l^-1^) in St.2 and minimum density (110 l^-1^) at station St.4. Maximum density was recorded in S2 and minimum density at station S4 (Figure 3). Five different microzooplankton taxa such as Tintinnids, Heterotrophic dinoflagellates, Ciliates, Foraminifera and Copepoda (nauplii) were identified in this study. Tintinnids made larger contribution in St.1 to the total abundance (mean 35%) followed in order Cilliates (23%), Dinoflagellates (21%) and Copepoda nauplii (17%). Foraminifera occurred in low abundance and contributed only 4% to the total population. At stations St.2, St.3 and St.4 Tintinnids were dominant followed by Dinoflagellates, Cilliates, and Copepoda nauplii. Foraminifera contributed only 3-5% to the total microzooplankton population (Figure 4).

### Species composition

**Figure 5:**
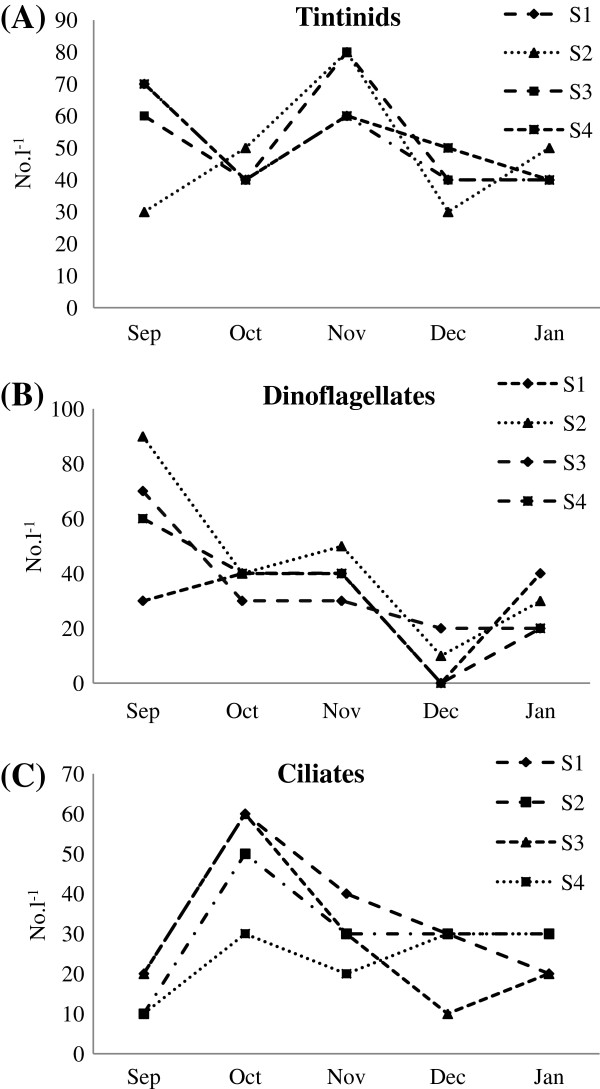
Variation in population density of Tintinnids, Dinoflagellates and Ciliates.

A total of 44 species belong to 19 genera of microzooplankton were identified during the study period (Tables 1 and 2). Tintinnids were the most dominant group in terms of number of species (Table 3) followed by Dinoflagellates. During November’11 Tintinids were recorded more at St.2 and St.3. Dinoflagellates were more during September’11 at St.2. Ciliates occured more during October’11 at almost all stations (Figure 5). The Tintinids were represented by *Amphorides* (1sp.), *Ascambelliella* (1 sp.), *Codonella* (1 sp.)*, Codonellopsis* (1 sp.)*, Eutintinmus* (3 spp.)*, Leprotintinnus* (1sp.)*, Metacylis* (1sp.), *Rhabdonella* (1 sp.) *Strenstrupiella* (1sp.), *Tintinidium* (1 spp.), and *Tintinnopsis* (6 spp.). Heterotrophic dinoflagellates were represented by *Noctiluca* (1 sp.), *Ornithocerus* (1 sp.), *Protoperidium* (8 sp.). Ciliates were represented by *Halteria* (1 sp.), *Lohmaniella* (2 sp.), *Strombidium* (2 sp.).

**Table 1:** Occurrence list of microzooplankton species in the polluted waters of Port Blair, South Andaman Island

**Species**	**September’11**	**October**	**November**	**December**	**January’12**
***Tintinnids***					
*Amphorella sp,* Daday			*	*	
*Amphorides pochytoecus,* Claparede		*			
*Ascambelliella sp,* Kofoid				*	*
*Codonella nationalis,* Brandt	*	*			*
*Codonellopsis ostenfeldi,* Schmidt	*		*		*
*Eutintinnus fraknoi,* Daday			*	*	
*Eutintinnus lusus undae,* Entz		*	*		
*Eutintinus tineus,* Zacherias	*		*	*	*
*Leprotintinnus nordquisti,* Brandt			*	*	
*Metacylis jorgenseni,* Cleve		*	*		
*Rhabdonella sp, Brandt*	*				
*Parundella caudata,* Ostenfeld				*	
*Streenstrupiella sp,* Balech	*	*			
*Tintinnidium primitivum,* Schmidt	*		*		
*Tintinnopsis beroidea,* Hada					*
*Tintinnopsis cylindrica,* Daday	*	*	*	*	
*Tintinnopsis glans,* Merkle		*		*	
*Tintinnopsis incertum,* Stein		*	*	*	*
*Tintinnopsis kofoidi,* Hada			*		*
*Tintinnopsis minuta,* Wailes	*				
**Heterotrophic dinoflagellates**					
*Noctiluca scintillans,* Kofoid		*			
*Ornithocerus magnificus,* Stein			*		
*Protoperidinium breve,* Paulsen	*	*	*	*	
*Protoperidinium brevipes,* Balech	*				
*Protoperidinium crassipes,* Kofoid			*		
*Protoperidinium depressum,* Bailey	*				*
*Protoperidinium divergens,* Ehrenberg	*				*
*Protoperidinium globulus,* Dangeard		*	*		
*Protoperidinium granii.* Ostenfeld	*				
*Protoperidinium heteracanthum,* Dangeard		*			
*Protoperidinium latistriatum,* Balech			*		*
*Protoperidinium nipponicum,* Bergh	*		*		*
*Protoperidinium ovatum,* Pouchet	*		*		
*Protoperidinium pellucidum,* Bergh	*		*		
*Protoperidinium stenii,* Jorgensen			*		
*Protoperidinium tuba,* Schiller	*				
***ciliates***					
*Halteria chlorelligera,* Khal			*		
*Lohmaniella spiralis,* Leegaard		*			
*Lohmaniella oviformes,* Leegaard		*	*	*	*
*Strombidium conicum,* Lohmann			*	*	*
*Strombidium minimum,* Gruber	*	*			*
***Copepod*nauplius**	*	*	*	*	*
**Foraminifera**		*		*	*

**Table 2:** Occurrence list of microzooplankton species in the relatively less polluted of Port Blair, South Andaman Island

**Species**	**September’11**	**October**	**November**	**December**	**January’12**
***Tintinnids***					
*Codonella nationalis,* Brandt	*		*		*
*Codonellopsis ostenfeldi,* Schmidt	*				
*Eutintinnus lusus undae,* Entz					*
*Eutintinus tineus,* Zacherias	*	*	*	*	*
*Leprotintinnus nordquisti,* Brandt			*		
*Metacylis jorgenseni,* Cleve		*	*		*
*Rhabdonella* sp, Brandt	*				
*Streenstrupiella sp,* Balech	*	*			
*Tintinnidium primitivum,* Schmidt	*		*		
*Tintinnopsis beroidea,* Hada	*				
*Tintinnopsis cylindrica,*Daday	*	*	*	*	*
*Tintinnopsis incertum,*Stein		*	*	*	*
*Tintinnopsis kofoidi,* Hada			*		
*Tintinnopsis minuta,* Wailes	*				
**Heterotrophic dinoflagellates**					
*Noctiluca scintillans,* Kofoid		*			
*Protoperidinium breve,* Paulsen	*	*	*		
*Protoperidinium brevipes,* Balech	*				
*Protoperidinium crassipes,* Kofoid			*		
*Protoperidinium depressum,* Bailey	*	*			*
*Protoperidinium divergens,* Ehrenberg	*	*			
*Protoperidinium globulus,* Dangeard			*		
*Protoperidinium latistriatum,*Balech				*	*
*Protoperidinium nipponicum,* Bergh			*	*	*
*Protoperidinium oblongum,* Aurivillius	*				
*Protoperidinium ovatum,* Pouchet	*		*		
*Protoperidinium tuba,* Schiller	*				
***Ciliates***					
*Halteria chlorelligera,* Khal			*		
*Lohmaniella spiralis,* Leegaard		*			
*Lohmaniella oviformes,* Leegaard		*	*	*	*
*Strombidium conicum,* Lohmann				*	*
*Strombidium minimum,* Gruber	*	*			*
***Copepod*nauplius**	*	*	*	*	*
**Foraminifera**	*	*	*		

**Table 3:** List of dominant species of Microzooplankton in polluted and relatively less polluted water of Port Blair

**Month**	**Area**	**Station**	**Number of Species**	**Dominant Taxa**
September’11	PW	S1	7	Tintinnids
		S2	7	Heterotrophic Dinoflagellates
	RPW	S3	6	Tintinnids
		S4	6	Tintinnids
October	PW	S1	5	Tintinnids
		S2	6	Tintinnids
	RPW	S3	4	Tintinnids
		S4	4	Tintinnids
November	PW	S1	6	Tintinnids
		S2	9	Tintinnids
	RPW	S3	8	Tintinnids
		S4	6	Tintinnids
December	PW	S1	4	Tintinnids
		S2	5	Tintinnids
	RPW	S3	3	Tintinnids
		S4	3	Tintinnids
January’12	PW	S1	3	Tintinnids
		S2	5	Tintinnids
	RPW	S3	3	Tintinnids
		S4	3	Tintinnids

PW: Polluted waters (S1 & S2); CW: Relatively polluted waters (S3 & S4).

### Species diversity

**Figure 6:**
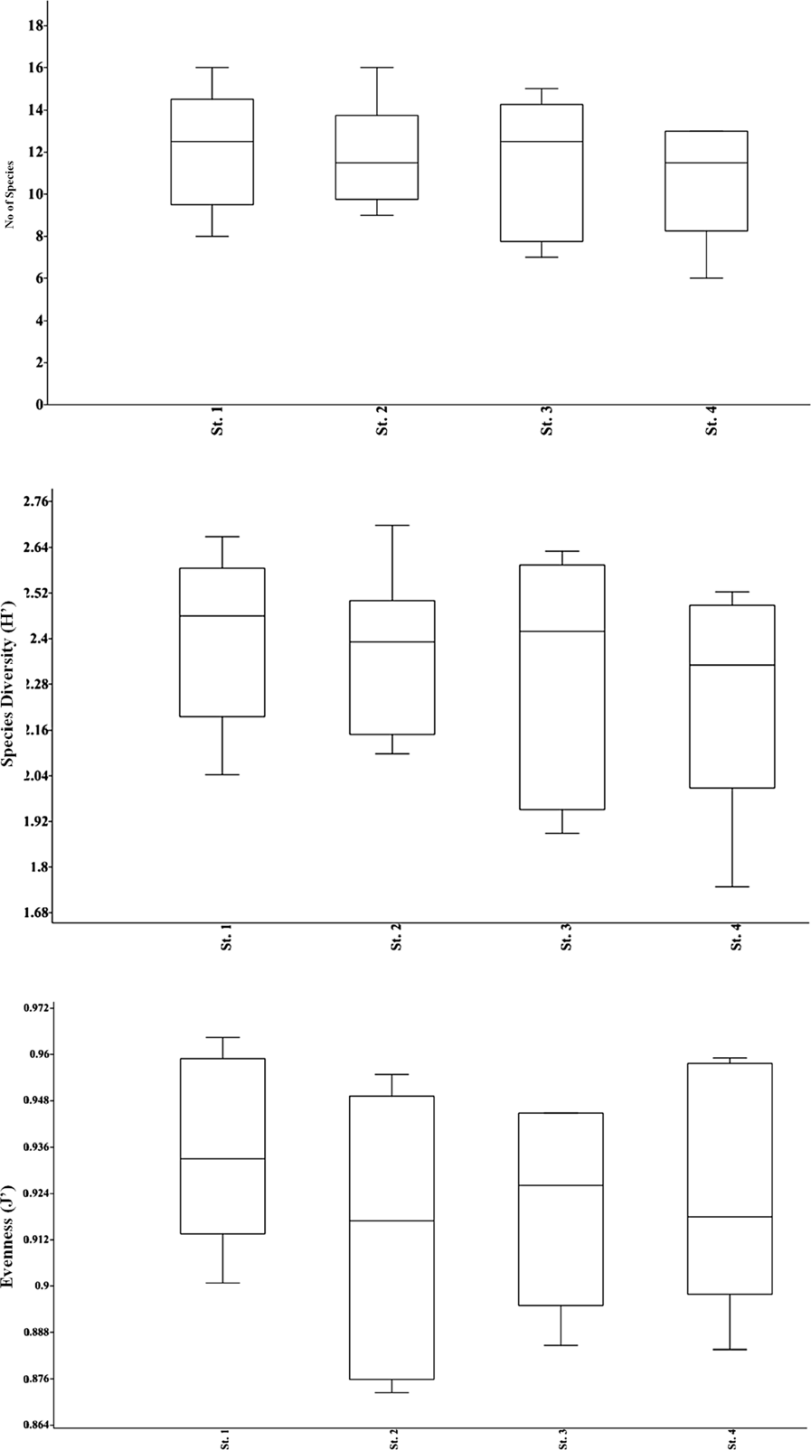
No of species (S), diversity indices (Shannon-Weiner index H’) and Evenness.

The number of species (S) and range of diversity indices in the study area are shown (Figure 6). The maximum number of species (33) recorded at stations St.1 and St.2 and minimum (28) obtained at St.3 during September. Relatively higher species diversity (H’ = 3.2) and equitability (J = 0.9) was found in polluted harbour area (St.1) and low diversity (H’ = 3.0) and lower equitability (J = 0.8) in microzooplankton population was recorded at station (St.4).

**Figure 7:**
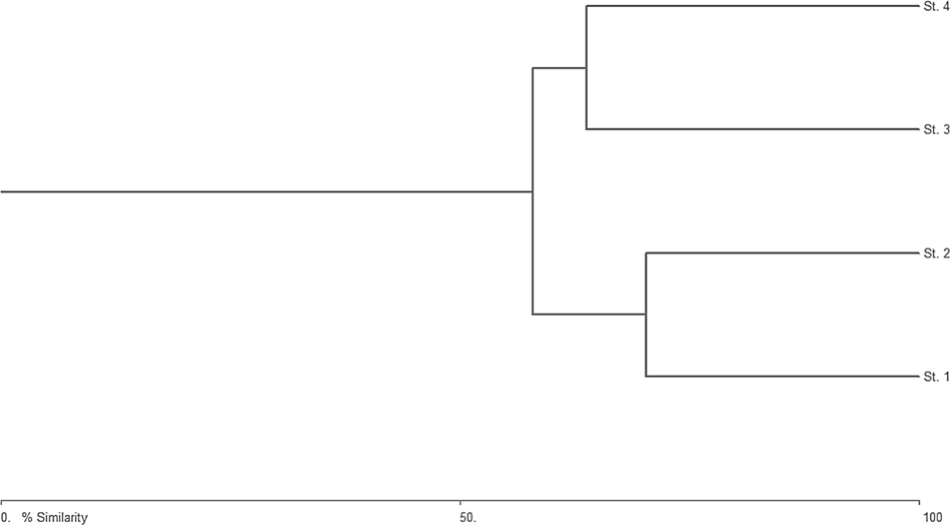
Bray Curtis similarity showing formation of groups in the study area.

Two separate assemblages of species were observed (Figure 7). The species in the St.1 and St.2 which is polluted formed one cluster in which mostly the tintinnids and dinoflagellates were dominant, and species in the relatively polluted St.3 and St.4 formed a separate cluster where Tintinnids and Ciliates were dominant.

## Discussion

This study is the first report on Microzooplankton from the coastal waters of Port Blair, South Andaman. Microzooplankton density varied from 160–350 l^-1^ in the study area. Maximum density (350 l^-1^) obtained at St.2 during November when temperature was high (28°C) and low density (160 l^-1^) at St.1 and St.4 during December at low temperature (24.8°C). Tintinnids which contributed substantially to the total density were also high at St.2 and St.3 (80 l^-1^) during November where the temperature was high. The concentration of Chlorophyll *a* and Tintinnid showed a reverse distribution in this study. Low density of Tintinnid (30 l^-1^) was observed in September’11 (St.2) when diatom bloom (*Coscinodiscus*) was noted could be due to inability of microzooplankton to graze on large-cell phytoplankton of harbour sample [11]. Similar observation has been reported earlier from the East China Sea [12]. A consistent increase in population size of Tintinnids was noticed from October onwards at almost all the stations (St.2 and St.3) and reached its peak in November (80 l^-1^) which in concomitant increase of salinity and temperature. Preference to particular environmental factors like temperature and salinity might have influenced their distribution. In this study, both temperature & salinity appear to control the abundance of tintinnids which also might affect their life cycle [13]. Tintinnids were represented by 20 species belong to 13 genera which is low compared to an earlier study from Bahuda estuary along the east coast of India [14]. Further, absence of four genera of tintinnids, viz., *Favella*, *Helicostomella Steenosemella*[13,15] and *Coxliella*[16] in this study could be due to the limited sampling season or according to their ecological distribution type of these organisms.

Dinoflagellates were represented by 17 species belong to 3 genera such as *Noctiluca*, *Ornithocerus Protoperidium* which is quite low compared to an earlier study from Oceanic region of Bay of Bengal and Andaman Sea [8] ascertained their oceanic preference. In this study maximum density of dinoflagellates was recorded during September at St.2 (90 l^-1^) followed by St.3 (70 l^-1^) when the dissolved oxygen, salinity and temperature values obtained low and ciliate population dwindled. These organisms are might be able to withstand or overcome the fluctuations in the environmental condition and possibly have a better chance of survival [8]. Most of the dinoflagellates are mixotrophic or heterotrophic and gain their nutrition through a combination of photosynthesis and uptake of dissolved or particulate organic material or phagotrophy on ciliates [17,18].

Ciliates comprised 5 species belong to 3 genera showed similar pattern of distribution as that of Tintinnids and were recorded high at St.1 and St.3 during October when the temperature and salinity were recorded high. Both Tintinnids and Ciliates are important phytoplankton grazers and are capable of exploiting pico- and nanoplankton, which are abundant in any marine coastal and estuarine systems and which cannot be utilized fully by the larger meso- and macrozooplankton. These smaller-size microzooplankton is ubiquitous and play an important role as a trophic link between pico- and nanoplankton and meso-and macro-metazoan predators and fishes in range of marine environments [19]. Foraminifera which are generally “benthic assemblage” were numerically less (avg 4%). Similar observation has been reported earlier from this area [8].

There exists a link between the environmental parameters and abundance of microzooplankton, hence the change of environmental variability during the study period affected the diversity and distribution pattern of microzooplankton. More intensive studies on seasonal variation of microzooplankton in relation to phytoplankton availability should be carried out from this area to understand the species composition and distribution pattern of microzoplankton. The diversity of Microzooplankton appears to relay more on resources than physical structure of the environment [20]. There are only a few studies on the feeding relationships between these taxa reported [21,22]. Studies on the different modes of nutrition and link between the microbial and classic planktonic food webs in the marine ecosystems should be carried out to understand their ecological significance.

## Methods

### Sampling area

**Figure 8:**
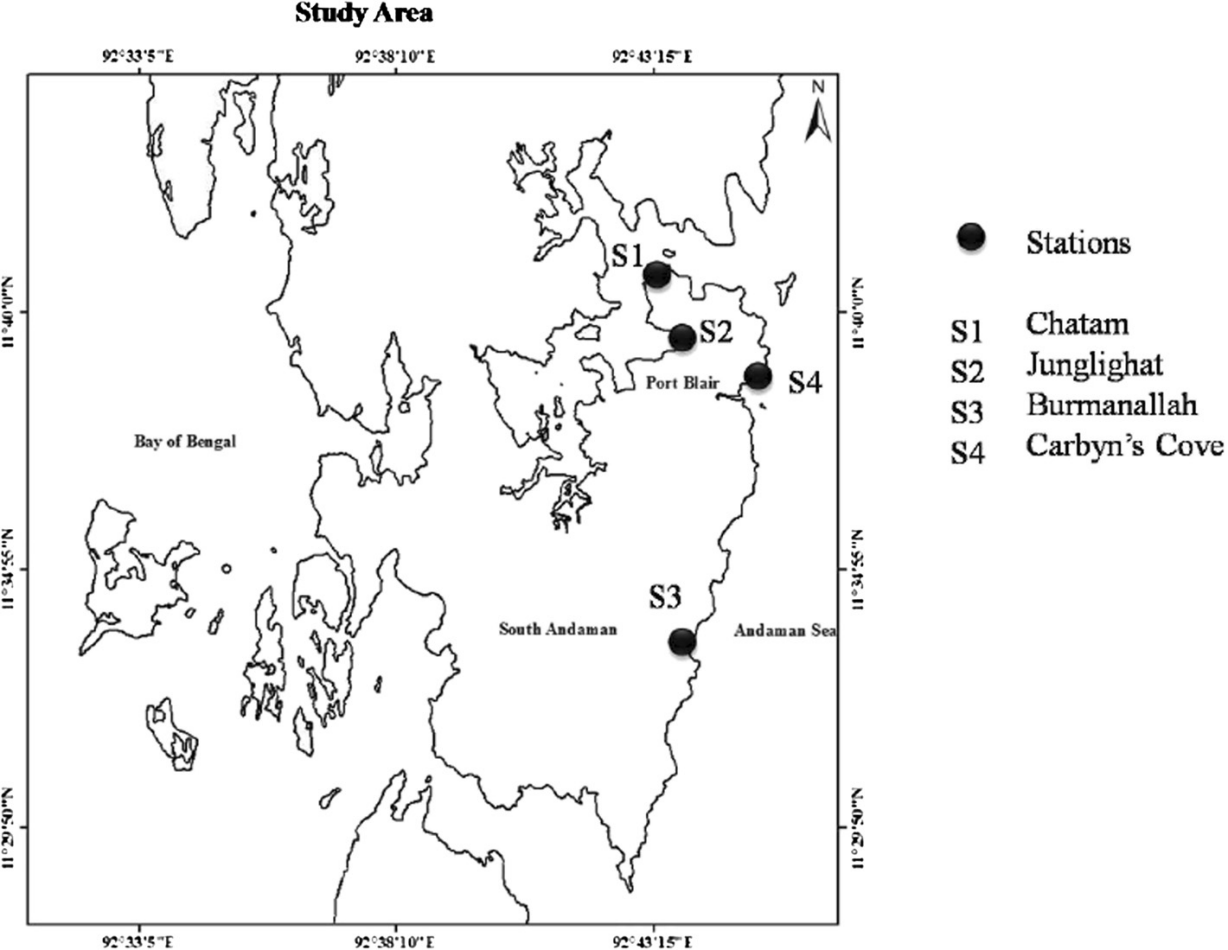
Location of sampling sites.

Microzooplankton study was carried out during September 2011 to January 2012 in two distinct areas viz., Polluted area i.e. Harbor & fish landing area (St. 1 and St. 2) whereas, St. (3 and 4) are relatively less polluted (Figure 8). Physicochemical parameters such as Seawater temperature, Salinity and Dissolved oxygen were recorded. Salinity was estimated with the help of a hand – held Refractometer (ATAGO). Dissolved Oxygen was estimated by the modified Winkler’s method and Phytoplankton biomass as chlorophyll *a* was estimated *Chlorophyll-a* (90% acetone method) spectrophotometrically in the laboratory [23] and is expressed as μg/L. Subsurface Seawater was filtered through a 200 μm plankton net and collected in a bucket. Further, this filtered water was then slowly passed through a 20 μm net. 1 liter of filtered Sea water was preserved in 1% Acid Lugol’s solution at all stations. The samples were left to settle for 24 hrs and concentrated to 10 ml by siphoning out the supernatant [7]. For Microzooplankton taxonomy studies, 1 ml sample was taken from concentrated sample by using a Sedgwick-Rafter counting chamber and examined under the plankton inverted microscope. Phytoplankton biomass as chlorophyll *a* was estimated [23]. The diversity indices were calculated [24].

## Competing interest

The authors declare that they have no competing interests.

## Authors’ contributions

SE has collected, analysed and identified the samples of microzooplankton. AK has collected and analysed the water samples. Phytoplankton data were collected and analysed with the assistance of KR. SSR has made statistical interpretation of data. JR performed design and analysis of data. PG has drafted the manuscript. All authors read and approved the final manuscript.
